# The transcription regulator ATF4 is a mediator of skeletal muscle aging

**DOI:** 10.1007/s11357-023-00772-y

**Published:** 2023-04-04

**Authors:** Matthew J. Miller, George R. Marcotte, Nathan Basisty, Cameron Wehrfritz, Zachary C. Ryan, Matthew D. Strub, Andrew T. McKeen, Jennifer I. Stern, Karl A. Nath, Blake B. Rasmussen, Andrew R. Judge, Birgit Schilling, Scott M. Ebert, Christopher M. Adams

**Affiliations:** 1 Division of Endocrinology, Diabetes, Metabolism and Nutrition, Departments of Medicine and Biochemistry and Molecular Biology, Mayo Clinic, 200 First Street SW, Rochester, MN 55905 USA; 2 University of Iowa, Iowa City, IA USA; 3 Buck Institute for Research on Aging, Novato, CA USA; 4 National Institute on Aging, NIH, Baltimore, MD USA; 5 University of Texas Medical Branch, Galveston, TX USA; 6 University of Florida, Gainesville, FL USA; 7 Emmyon, Inc., Rochester, MN USA; 8 Iowa City Veterans Affairs Medical Center, Iowa City, IA USA

**Keywords:** Aging, Sarcopenia, Skeletal muscle, Skeletal muscle atrophy, ATF4, Protein turnover

## Abstract

Aging slowly erodes skeletal muscle strength and mass, eventually leading to profound functional deficits and muscle atrophy. The molecular mechanisms of skeletal muscle aging are not well understood. To better understand mechanisms of muscle aging, we investigated the potential role of ATF4, a transcription regulatory protein that can rapidly promote skeletal muscle atrophy in young animals deprived of adequate nutrition or activity. To test the hypothesis that ATF4 may be involved in skeletal muscle aging, we studied fed and active muscle-specific ATF4 knockout mice (ATF4 mKO mice) at 6 months of age, when wild-type mice have achieved peak muscle mass and function, and at 22 months of age, when wild-type mice have begun to manifest age-related muscle atrophy and weakness. We found that 6-month-old ATF4 mKO mice develop normally and are phenotypically indistinguishable from 6-month-old littermate control mice. However, as ATF4 mKO mice become older, they exhibit significant protection from age-related declines in strength, muscle quality, exercise capacity, and muscle mass. Furthermore, ATF4 mKO muscles are protected from some of the transcriptional changes characteristic of normal muscle aging (repression of certain anabolic mRNAs and induction of certain senescence-associated mRNAs), and ATF4 mKO muscles exhibit altered turnover of several proteins with important roles in skeletal muscle structure and metabolism. Collectively, these data suggest ATF4 as an essential mediator of skeletal muscle aging and provide new insight into a degenerative process that impairs the health and quality of life of many older adults.

**Supplementary Information:**

The online version contains supplementary material available at 10.1007/s11357-023-00772-y.

## Introduction

Age-related skeletal muscle atrophy, also known as sarcopenia, is a slowly progressive process that can be debilitating for many people. In most people, early effects of muscle aging emerge in the fourth decade of life, when muscle strength begins to decline. Over the ensuing decades, both strength and muscle mass decline, however, strength is lost more rapidly than muscle mass, and thus, a reduction in muscle quality (strength per unit muscle mass) is a central feature of skeletal muscle aging [1, 2]. By the seventh decade of life, many people have overt skeletal muscle atrophy, and almost all people are significantly weaker than they were as young adults. Consequences of muscle aging can include frailty, impaired activity, falls, and loss of independent living.

Although skeletal muscle aging has a significant impact on health and quality of life, its molecular mechanisms are complex, challenging, and not well understood [3–5]. In previous studies, we investigated the potential role of ATF4, a stress-inducible transcription regulator in the basic leucine zipper (bZIP) superfamily [6, 7]. In skeletal muscle fibers of young adult mice (3 months old), forced expression of ATF4 is sufficient to induce skeletal muscle atrophy within one week [8]. Conversely, 3-month-old muscle-specific ATF4 knockout mice (ATF4 mKO mice) are partially protected from rapid, acute skeletal muscle atrophy during starvation and immobilization [9, 10]. Furthermore, at an old age (22 months), ATF4 mKO mice have greater strength and muscle mass than littermate control mice [11]. The previously observed phenotype of 22-month-old ATF4 mKO mice suggested that ATF4 may be required for age-related muscle atrophy and weakness; however, the study was limited by an absence of data comparing ATF4 mKO mice and littermate controls at 6 months of age, after development of peak muscle mass and strength but prior to the onset of age-related skeletal muscle atrophy and weakness. Thus, we could not rule out the possibility that ATF4 mKO mice simply had greater muscle mass and function throughout middle and old age. For perspective, 6-month-old mice are considered to be at a similar life phase as 30-year-old humans, and 22-month-old mice are considered to be at a similar life phase as 65-year-old humans [12].

In the current study, we tested the hypothesis that ATF4 may play an essential role in the loss of muscle mass and function that occurs with normal aging. To that end, we compared the phenotypes of ATF4 mKO mice and littermate control mice at both 6 and 22 months of age. In addition, because skeletal muscle aging involves significant alterations in skeletal muscle gene expression and protein metabolism, which are thought to contribute to the phenotypes of skeletal muscle aging [3, 13], we used unbiased transcriptomic and proteomic methods to test the hypothesis that ATF4 might be responsible for specific age-related molecular changes in skeletal muscle.

## Methods

### Mouse strains and protocols

ATF4 mKO mice were described previously [9] and were generated by crossing mice homozygous for a floxed *ATF4* allele (*ATF4 L/L*) to *ATF4 L/L* mice heterozygous for a muscle creatine kinase (*MCK*)-*Cre* transgene. Control mice were *ATF4 L/L* littermates that lacked the *MCK-Cre* transgene. All mice in this study were males on a C57BL/6 background. Different cohorts of 22-month-old mice were used for different experiments, but in each experiment, the 22-month-old control mice were littermates of the 22-month-old ATF4 mKO mice. Female mice were not studied due to cost considerations and economic limitations. Mice were housed (up to five mice per cage) in ventilated cages (Thoren Rack system, no. 9 size cages) at 21 °C with 12:12-h light/dark cycles and ad libitum access to food and water. In all but the protein turnover studies, discussed below, the diet was Harlan Teklad formula 7913. Water was obtained from a filtered automatic watering system. The colony was confirmed to be specific pathogen-free via routine biannual testing of sentinel mice for a wide range of pathogens including mouse hepatitis virus, Parvovirus (minute virus of mice, mouse parvovirus), Theiler’s murine encephalomyelitis virus, mouse rotovirus (EDIM), Sendai, Mycoplasma pulmonis, murine norovirus, pneumonia virus of mice, Reo3, Ectromelia, mouse adenovirus 1 and 2, lymphocytic choriomeningitis virus, pinworms, fur mites, ectoparasites, and endoparasites. The mouse housing room contained other strains of mice of both sexes. Forelimb grip strength was determined using a grip strength meter equipped with a triangular pull bar (Columbus Instruments), as described previously [11, 14]. Ex vivo muscle force generation was determined using an Aurora Scientific 1200A Intact Muscle Test System to determine maximal and specific tetanic force in isolated extensor digitorum longus muscles, as described previously [15]. The extensor digitorum longus (EDL) muscle was used for specific force measurements because it is a small muscle that can be adequately oxygenated ex vivo, which is essential for accurate measurement of contractile properties [16]. In the current study, EDL muscles were used for specific force measurements and were not collected for other analyses. The grip strength data and specific force data from 22-month-old control, and ATF4 mKO mice were taken from our previous study of 22-month-old control and ATF4 mKO mice [11]. All of the other data in the current study were previously unpublished. Endurance exercise capacity was determined using a motor-driven open treadmill with a shock grid (Columbus Instruments), as described previously [15, 17]: For 2 days, mice were acclimated to running on a motor-driven open treadmill with a shock grid (Columbus Instruments) for 5 min/day. During acclimation, the treadmill speed was set at 5 m/min, and the treadmill incline was set at 0%. On the third day, exercise tolerance was tested, the shock grid was set at 0.2 mA, and the treadmill incline was set at 10%. For the first 5 min of testing, treadmill speed was set at 5 m/min. Every 2 min thereafter, the treadmill speed was increased by 2 m/min. Running was terminated when mice contacted the shock grid for 10 s. For analyses of muscle mass, we used two of the largest limb muscles, gastrocnemius, and quadriceps, and we chose to use large muscles because, relative to smaller muscles, they make a larger contribution to overall muscle mass. Quadriceps were used for proteomic analyses based on similar considerations and based on a requirement for a large amount of tissue for the proteomic method used in this study. The transcriptomic analyses used an intermediate-sized muscle, the tibialis anterior (TA), based on our experience with transcriptomic analyses in the TA and so that the data from this study can be used for comparative transcriptomic studies of mouse TA muscles across a variety of experimental conditions. Cage side observations of all mice were made daily throughout the study. Aggressive male mice were separated from their cage mates and housed individually; there was no difference in aggression between the strains. Euthanasia was performed by subjecting animals to CO2 exposure (flow rate of 3 L/min) until breathing stopped for a period of 1 min, and euthanasia was confirmed by decapitation. Euthanasia methods were approved by the Panel on Euthanasia of the American Veterinary Medical Association. All animal procedures were approved by the Institutional Animal Care and Use Committee of the University of Iowa.

### Histological analysis

Harvested muscles were embedded in Tissue Freezing Medium (Triangle Biomedical Sciences), and a Microm HM 505E cryostat was used to prepare 10-μm sections from the muscle midbelly. Cryosections were blocked for 1 h at 25 °C in 5% normal goat serum (NGS) and then incubated for 2 h at 25 °C in 5% NGS containing a 1:2000 dilution of anti-laminin (Sigma L9393). Sections were then rinsed with PBS, incubated for 1 h at 25 °C in 5% NGS containing a 1:2000 dilution of Alexa Fluor 568-conjugated anti-rabbit IgG and then mounted in Vectashield (Vector Laboratories). Muscle sections were examined and photographed with a Nikon Eclipse Ti automated inverted microscope equipped with NIS-Elements BR digital imaging software. Image analysis was performed with ImageJ and MyoVision software [18], and muscle fiber diameter was determined with the lesser diameter (minimal Feret diameter) method, as described previously [17].

### RNA sequencing

Mouse tibialis anterior muscle RNA was extracted using TRIzol solution (Invitrogen) and purified using the RNeasy kit and RNase-free DNase Set (Qiagen) according to the manufacturer protocol. Samples were quantified using the Trinean DropSense 16, and RNA quality was assessed using the Agilent BioAnalyzer, with all samples showing an RNA integrity Number (RIN) greater than 8. Libraries were prepared using the TruSeq Stranded mRNA Sample Prep Kit (Illumina). Briefly, oligo-dT purification of polyadenylated RNA was followed by reverse transcription, fragment purification, end polishing and ligation to indexed adaptors. Paired-end sequencing with a read length of 2 × 150 bp was performed on the Illumina platform at the Genomics Division of the Iowa Institute of Human Genetics.

### Differential mRNA expression and gene set enrichment analysis

Sequencing reads were trimmed to remove adaptor sequences using Trimmomatic [19] and mapped to the *M. musculus* reference genome mm10 using RNA-star (v2.7.8a) [20]. FeatureCounts (v2.0.1) was used to count the number of reads uniquely mapping to annotated genes [21] and was used for normalization and differential gene expression analysis using DESeq2 (v2.11.40.7) [22] on the online platform Galaxy [23]. Significantly differentially expressed transcripts were identified as those with a FDR adjusted *P* value < 0.1. Gene set enrichment analysis (GSEA) was performed for gene sets from the Reactome database (v7.5.1) using GSEA v4.2.3 [24, 25]. LFC was used to rank the genes for analysis. Significantly enriched gene sets were identified as those with a FDR < 0.25. Additionally, a difference in nominal enrichment score > 0.4 between genotypes was used to identify gene sets enriched in control muscle aging, but not ATF4 mKO, muscle aging.

### Chemicals

Deuterated leucine (> 98% stable isotope) was obtained from Cambridge Isotope Labs. Acetonitrile (#AH015) and water (#AH365) were from Burdick & Jackson (Muskegon, MI). Iodoacetamide (IAA, #I1149), dithiothreitol (DTT, #D9779), formic acid (FA, #94318-50ML-F), and triethylammonium bicarbonate buffer 1.0 M, pH 8.5 (#T7408), were from Sigma Aldrich (St. Louis, MO); urea (#29700) was from Thermo Scientific (Waltham, MA); sequencing grade trypsin (#V5113) was from Promega (San Luis Obispo, CA); and HLB Oasis SPE cartridges (#186003908) were from Waters (Milford, MA).

### Protein turnover studies and mass spectrometric analysis

At 19.5 months of age, cohorts of control and ATF4 mKO mice were switched from standard chow (Harlan-Teklad formula 7913) to “Amino Acid Defined” chow (Envigo TD.99366), which contains 11.1 g/kg leucine. Ten weeks later, at 22 months of age, mice were switched to a modified Envigo TD.99366 diet that contained 11.1 g/kg deuterated leucine ([5,5,5-^2^H_3_]-L-leucine) in place of unlabeled leucine. Quadriceps muscles (3-5 per genotype and time point) were collected after 3, 7, 15, or 30 days on the deuterated leucine diet and then stored in liquid nitrogen. As described in great detail in the Supplemental Experimental procedures (Supplemental Experimental Procedures), tissues were homogenized using a TissueLyzer II (Qiagen, Hilden, Germany), followed by lysis, reduction and alkylation, and proteolytic digestion using sequencing grade trypsin (Promega, San Luis Obispo, CA) at a 1:25 enzyme:substrate ratio (wt/wt). Samples were analyzed by reverse-phase HPLC-ESI-MS/MS using the Eksigent Ultra Plus nano-LC 2D HPLC system (Dublin, CA) combined with a cHiPLC system directly connected to an orthogonal quadrupole time-of-flight SCIEX TripleTOF 6600 mass spectrometer (SCIEX, Redwood City, CA) (for details, see Supplemental Experimental Procedures).

### Quantitative analysis of abundance and turnover

For calculation of protein abundance changes, data-independent acquisitions (DIA) from six samples (three ATF4 mKO and three littermate control samples) were quantitatively processed using Spectronaut v14 (14.7.201007) software from Biognosys (Schlieren, Switzerland). Experimental parameters for the Spectronaut processing are provided in the supplemental methods document (Supplemental Experimental Procedures).

Precursor-pool corrected protein turnover rates were calculated in R using the TurnoveR tool, previously described in detail [26]. The TurnoveR pipeline is similar to analytical approaches employed in previous studies using the Topograph software platform [27–29]; additional details are also described in the supplemental methods (Supplemental Experimental Procedures). Briefly, precursor pool isotopic enrichments, fractional abundances of newly synthesized proteins, and half-lives were calculated for each protein using the TurnoveR tool. The precursor pools did not significantly change between treatment groups but increased over time points with the incorporation of heavy leucine from the diet (Fig. 6B). The distribution of newly synthesized proteins did not significantly differ between ATF4 mKO and littermate control muscles but increased over time as expected (Fig. 6C–D). This yielded the protein turnover rates that were transformed into half-lives using a simple conversion [half-life = -ln(2)/turnover]. For statistical comparison of turnover rates between ATF4 mKO and control samples, first-order equations were natural log transformed, making a linear relationship between the log of percent newly synthesized proteins and time. Then, linear modeling statistics were applied to determine if the interaction between the log-transformed percent newly synthesized values and time are different between ATF4 mKO and control samples, and the *P* value of the difference in interaction was used to determine whether protein turnover rates were significantly different by genotype. To adjust for multiple hypotheses testing, *q* values (false discovery rate) was calculated using the Storey method [30] with the “qvalue” package in R. Adjusted *P* values were also calculated using the Benjamini-Hochberg correction. A full report of protein turnover rates, annotations, variance, statistical analysis, and other quantitative information is provided in Table S5.

### Statistical analysis

RNA-sequencing and protein turnover data were analyzed as described above. All other statistical analyses were performed with GraphPad Prism. The statistical tests and sample sizes are provided in the figure legends. 

### Data availability

Raw mass spectrometric data files, database search results, quantitative reports, spectral libraries, protein databases, and other supplementary files are available on MassIVE (MSV000088083) and ProteomeXchange (PXD028444). All code used for turnover analysis is freely available on GitHub (https://github.com/CameronWehrfritz/Adams-Protein-Turnover-Paper.git). RNA-seq data are deposited in Gene expression omnibus (GEO) (GEO accession no.: GSE212675).

## Results

### ATF4 expression in skeletal muscle fibers contributes to age-related declines in skeletal muscle strength, muscle quality, and endurance exercise capacity

To better understand the effects of ATF4 in skeletal muscle aging, we investigated muscle-specific ATF4 knockout (ATF4 mKO) mice, which have a lifelong absence of ATF4 expression in skeletal muscle fibers due to the presence of homozygous floxed *ATF4* alleles and an *MCK-Cre* transgene, which excises floxed alleles in fully differentiated skeletal muscle fibers and heart, but not satellite cells [9–11, 31, 32]. We compared ATF4 mKO mice to littermate controls, which are also homozygous for the floxed *ATF4* allele but lack the *MCK-Cre* transgene. In both genotypes, we assessed strength, muscle quality (specific force), and endurance exercise capacity at 6 months of age, when mice have achieved peak muscle mass and function, and at 22 months of age, when mice begin to exhibit age-related deficits in skeletal muscle function [11]. The studies of strength and muscle quality build upon a previous study where we found that 22-month-old ATF4 mKO mice have greater grip strength and specific force than age-matched littermate control mice [11]. Importantly, the cohorts of 6- and 22-month-old control and ATF4 mKO mice did not differ in total body weight, which can influence muscle function and mass (Fig. S1A).

**Fig. 1:**
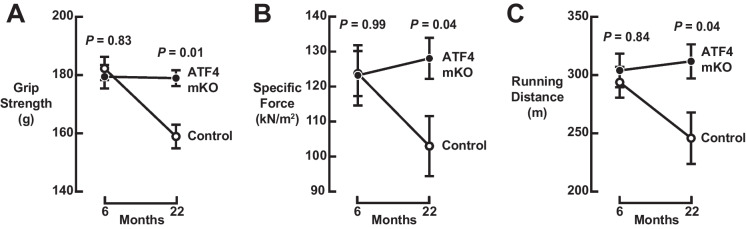
ATF4 promotes age-related declines in skeletal muscle strength, muscle quality, and endurance exercise capacity. Weight-matched cohorts of 6- and 22-month-old littermate control and ATF4 mKO mice were subjected to assessments of in vivo grip strength (**A**), ex vivo specific force (**B**), and in vivo treadmill running (**C**). Data are means ± SEM from ≥14 mice in (**A**), ≥ 7 mice in (**B**), and ≥ 13 mice in (**C**). Individual data points and body weight data are shown in Fig. S1. The grip strength and specific force data from 22-month-old control and ATF4 knockout mice were shown previously [11]. *P* values compare control and ATF4 mKO mice at each time point using two-way ANOVA with Šidák’s multiple comparisons test

At 6 months of age, ATF4 mKO and littermate control mice possessed equivalent grip strength, specific force, and exercise capacity relative to littermate controls (Figs. 1A–C and S1B–D), indicating that an absence of ATF4 expression in skeletal muscle fibers does not impair or enhance normal muscle function. As expected, control mice exhibited a decline in muscle function between 6 and 22 months, due to normal muscle aging (Figs. 1A–C and S1B–D). In contrast, ATF4 mKO mice were protected from age-related declines in muscle function and, thus, maintained grip strength, specific force, and exercise capacity between 6 and 22 months of age (Figs. 1A–C and S1B–D). These results indicate that ATF4 expression in skeletal muscle fibers plays an important role in the loss of skeletal muscle strength, muscle quality, and endurance exercise capacity between 6 and 22 months of age.

### ATF4 expression in skeletal muscle fibers contributes to age-related skeletal muscle atrophy

**Fig. 2:**
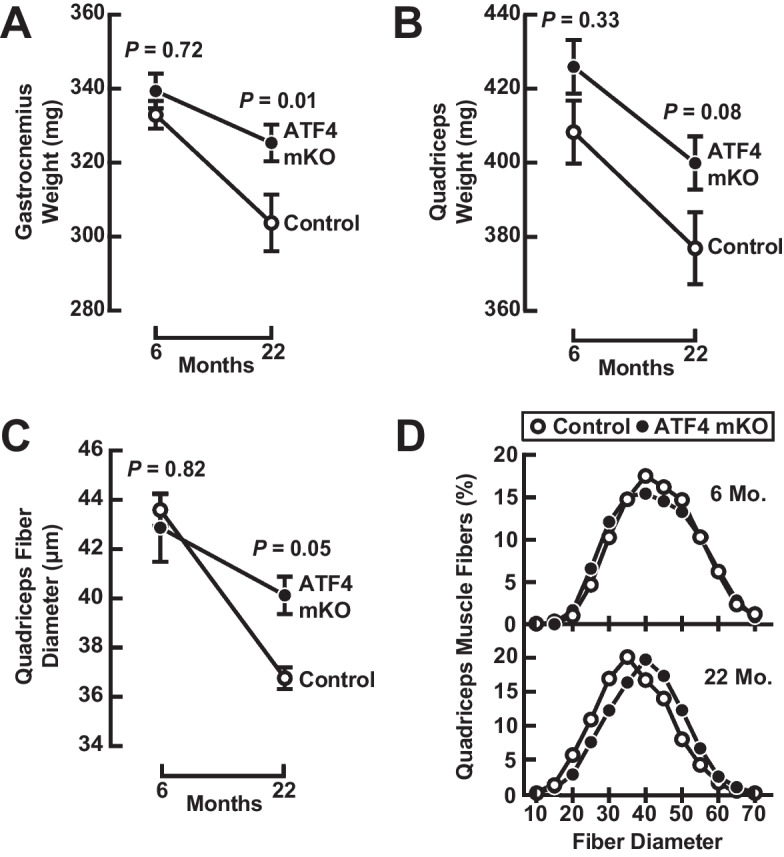
ATF4 promotes age-related skeletal muscle atrophy. Weight-matched cohorts of 6- and 22-month-old littermate control and ATF4 mKO mice were subjected to assessments of combined weight of bilateral gastrocnemius muscles (**A**), combined weight of bilateral quadriceps muscles (**B**), mean quadriceps muscle fiber diameter (**C**), and quadriceps muscle fiber size distribution of > 3950 muscle fibers per condition (**D**). Data are means ± SEM from ≥10 mice in (**A**), ≥ 10 mice in (**B**), and ≥ 5 mice in (**C**) and (**D**). Individual data points are shown in Fig. S2. *P* values compare control and ATF4 mKO mice at each time point using two-way ANOVA with Šidák’s multiple comparisons test

To test the hypothesis that ATF4 might be required for age-related skeletal muscle atrophy, we compared muscle weights and fiber diameter from ATF4 mKO mice and littermate controls. At 6 months of age, ATF4 mKO and control mice possessed similar muscle mass (Figs. 2A–B and S2A–B) and muscle fiber diameter (Figs. 2C–D and S2C), indicating that an absence of ATF4 expression in skeletal muscle fibers does not impair development of muscle mass or induce muscle hypertrophy. Between 6 and 22 months, both genotypes lost muscle mass and fiber size, due to age-related muscle atrophy (Figs. 2A–D and S2A–C). Importantly, however, ATF4 mKO mice exhibited less age-related muscle atrophy than control mice (Figs. 2A–D and S2A–C). Thus, a targeted reduction of ATF4 expression in skeletal muscle fibers partially prevents age-related skeletal muscle atrophy between 6 and 22 months of age.

### In skeletal muscle, ATF4 contributes to basal expression of 30 mRNAs involved in stress signaling and translational control

**Fig. 3:**
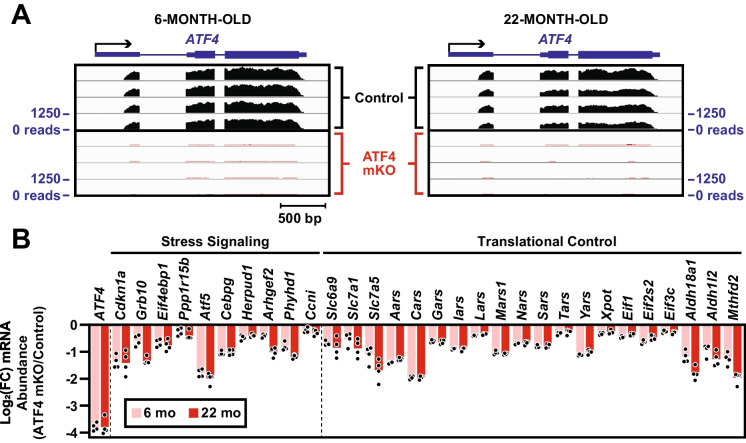
In skeletal muscle, ATF4 contributes to basal expression of 30 mRNAs involved in stress signaling and translational control. Skeletal muscle from 6- and 22-month-old control and ATF4 mKO mice was subjected to RNA sequencing (RNA-seq). **A** RNA-seq read alignments at the *ATF4* gene. The level of ATF4 mRNA in 22-month-old control muscle is 14% lower than the level of ATF4 mRNA in 6-month-old control muscle (FDR = 0.09). **B** mRNAs whose levels were significantly decreased (FDR < 0.1) in ATF4 mKO muscles (relative to age-matched control muscles) at both 6 and 22 months of age. Each data point represents one muscle, and bars indicate the average log2 fold-change

ATF4 is an essential subunit of at least seven different heterodimeric bZIP transcription factors that regulate gene expression in skeletal muscle fibers [6, 8, 33]. Thus, to begin to understand the preventive effects of *ATF4* gene deletion on age-related muscle atrophy and weakness, we used RNA sequencing (RNA-seq) to assess mRNA levels in skeletal muscle from both younger (6 months old) and older (22 months old) control and ATF4 mKO mice (Table S1). As expected, at 6 and 22 months of age, *ATF4* transcripts were abundant in littermate control muscles and nearly absent in ATF4 mKO muscles (Fig. 3A). In addition, at both time points, ATF4 mKO muscles had lower levels of 10 transcripts involved in stress signaling (*Cdkn1a/p21*, *Grb10*, *Eif4ebp1/4E-BP1*, *Ppp1r15b/CReP*, *ATF5*, *Cebpg/C-EBPγ*, *Herpud1*, *Arhgef2*, *Phyhd1*, and *Ccni/Cyclin I*), and 20 transcripts involved in translational control (*Slc6a9*, *Slc7a1*, *Slc7a5*, *Aars*, *Cars*, *Gars*, *Iars*, *Lars*, *Mars*, *Nars*, *Sars*, *Tars*, *Yars*, *Xpot*, *Eif1*, *Eif2s2*, *Eif3c*, *Aldh18a1*, *Aldh1l2*, and *Mthfd2*) (Fig. 3B). Of these 30 ATF4-dependent transcripts, all but two (*Phyhd1* and *Ccni*) arise from genes that are known to be directly activated by ATF4 heterodimers [10, 34–49]. Furthermore, *Cdkn1a/p21* and *Grb10* encode proteins that are known to negatively regulate skeletal muscle mass [10, 50–53]. As previously reported in liver-specific ATF4 knockout mice [54], ATF4 mKO muscles also contained two changes that are likely explained by local chromosome rearrangement following Cre-mediated excision of the *ATF4* allele: low level expression of a non-natural *ATF4-Cacna1i* fusion transcript and decreased levels of *Rps19bp1* mRNA, an endogenous transcript that is poorly expressed in control skeletal muscle and absent in ATF4 mKO muscles (Fig. S3). Interestingly, the transcripts that were significantly decreased in 6- and 22-month-old ATF4 mKO muscles were not regulated by aging in control muscles; in other words, these mRNAs did not significantly increase or decrease as littermate control mice aged from 6 to 22 months. These data identified primary molecular effects of *ATF4* gene deletion in skeletal muscle fibers, which occur before and after skeletal muscle phenotypes emerge. These primary effects include reduced basal expression of 30 ATF4 target genes involved in stress signaling and translational control.

### During skeletal muscle aging, ATF4 promotes induction of transcripts involved in inflammation, cellular senescence, and Rho GTPase signaling

**Fig. 4:**
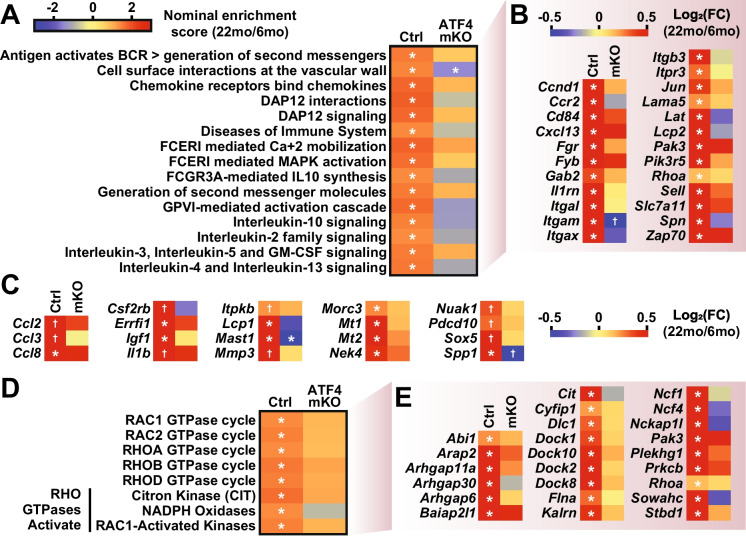
During skeletal muscle aging, ATF4 promotes induction of transcripts involved in inflammation, cellular senescence, and Rho GTPase signaling. RNA-Seq data from skeletal muscle of 6- and 22-month-old control and ATF4 mKO mice were used to identify pathways that were induced by aging in control but not ATF4 mKO muscles. **A–B** Inflammation-associated Reactome gene sets that were significantly induced by aging in control but not ATF4 mKO muscles (**A**) and key individual transcripts from those gene sets (**B**). **C** Additional senescence-associated mRNAs (from CellAge and SenMayo gene panels) that were induced by aging in control but not ATF4 mKO muscles. **D–E** Reactome Rho GTPase signaling gene sets that were significantly induced by aging in control but not ATF4 mKO muscles (**D**) and key transcripts from those gene sets (**E**). In (**A**) and (**D**), asterisks indicate FDR < 0.25. In (**B**), (**C**), and (**E**), asterisks indicate FDR < 0.05, and cross indicates FDR < 0.10

We hypothesized that that loss of ATF4 expression in skeletal muscle fibers might also have secondary effects that emerge between 6 and 22 months of age. As an initial test of that hypothesis, we performed gene set enrichment analysis (GSEA) of the RNA-Seq data and identified 36 Reactome gene sets that were induced by aging in control skeletal muscle and not significantly affected by aging in ATF4 mKO skeletal muscle (Fig. S4 and Table S2). Interestingly, 15 of these 36 gene sets were thematically linked around inflammation (Fig. 4A) and were composed of partially overlapping sets of transcripts involved in inflammation and the senescence-associated secretory phenotype (Fig. 4B). Consistent with this finding, 19 additional mRNAs contained in transcriptomic signatures of cellular senescence were significantly increased by aging in control muscle but not ATF4 mKO muscle (Figs. 4C [55, 56]), including two metallothioneins (*Mt1* and *Mt2*) that promote muscle atrophy and weakness [57]. Another 8 of the 36 gene sets induced by aging in control but not ATF4 mKO muscle were thematically linked around RhoGTPase signaling (Fig. 4D–E), which has been implicated in the control of muscle mass [58–60]. Altogether, 711 mRNAs were significantly induced by aging in control skeletal muscle but not ATF4 mKO muscle (Table S3), representing ~ 3% of total measured transcripts and ~ 20% of total transcripts induced by aging in control skeletal muscle. Thus, ATF4 expression in skeletal muscle fibers is required for age-related induction of transcripts involved in cellular processes such as inflammation, cellular senescence, and Rho GTPase signaling.

### During skeletal muscle aging, ATF4 promotes repression of transcripts involved in mitochondrial function, protein synthesis, and metabolism of amino acids, polyamines, glutathione, and nicotinamide

**Fig. 5:**
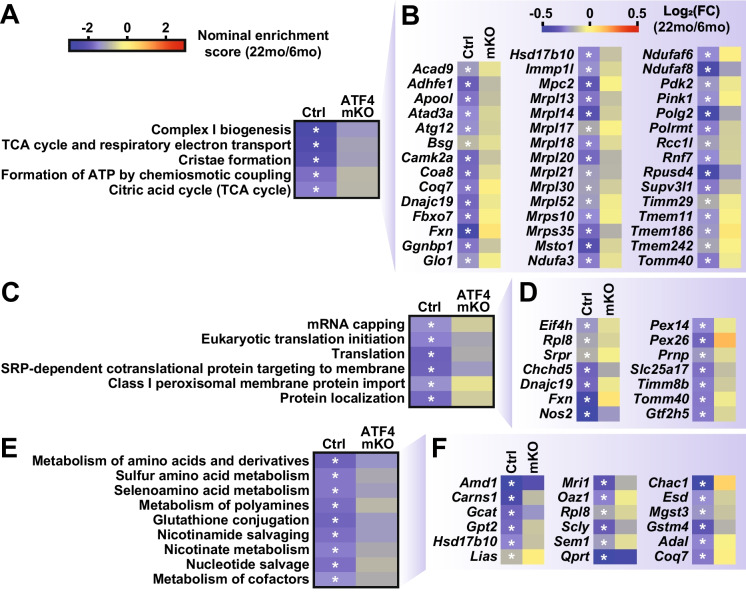
During skeletal muscle aging, ATF4 promotes repression of transcripts involved in mitochondrial function, protein synthesis, and metabolism of amino acids, polyamines, glutathione, and nicotinamide. RNA-Seq data from skeletal muscle of 6- and 22-month-old control and ATF4 mKO mice were used to identify Reactome gene sets that were repressed by aging in control but not ATF4 mKO muscles. **A–B** Mitochondria-related gene sets that were significantly repressed by aging in control but not ATF4 mKO muscles (**A**) and key individual transcripts from those gene sets (**B**). **C–D** Protein synthesis-related gene sets that were significantly repressed by aging in control but not ATF4 mKO muscles (**C**) and key transcripts from those gene sets (**D**). **E–F** Gene sets related to amino acid, polyamine, glutathione, and nicotinamide metabolism (**E**) and key transcripts from those gene sets (**F**). In (**A**), (**C**), and (**E**), asterisks indicate FDR < 0.25. In (**B**), (**D**), and (**F**), asterisks indicate FDR < 0.05

We next asked whether loss of ATF4 might prevent repression of cellular processes that normally decline during skeletal muscle aging. To that end, we identified 24 Reactome gene sets that significantly decreased with aging in control skeletal muscle but not in ATF4 mKO muscle (Fig. S5 and Table S2). Interestingly, most of these gene sets were thematically linked around cellular processes that are necessary for the maintenance of skeletal muscle mass and function, including mitochondrial function (Fig. 5A), protein synthesis (Fig. 5C), and metabolism of amino acids, polyamines, glutathione, and nicotinamide (Fig. 5E). Many transcripts involved in these cellular processes were strongly repressed by aging in control muscles, but their expression was maintained in aged ATF4 mKO muscles (Fig. 5B, D, and F). Altogether, 708 mRNAs were significantly repressed by aging in control skeletal muscle but not ATF4 mKO muscle (Table S4), representing ~ 3% of total measured transcripts and ~ 23% of transcripts repressed by aging in control skeletal muscle. These data indicate that ATF4 expression in skeletal muscle fibers may contribute to age-related repression of metabolic processes that are necessary to maintain healthy skeletal muscle mass and function.

### ATF4 influences turnover of specific proteins in skeletal muscle

**Fig. 6:**
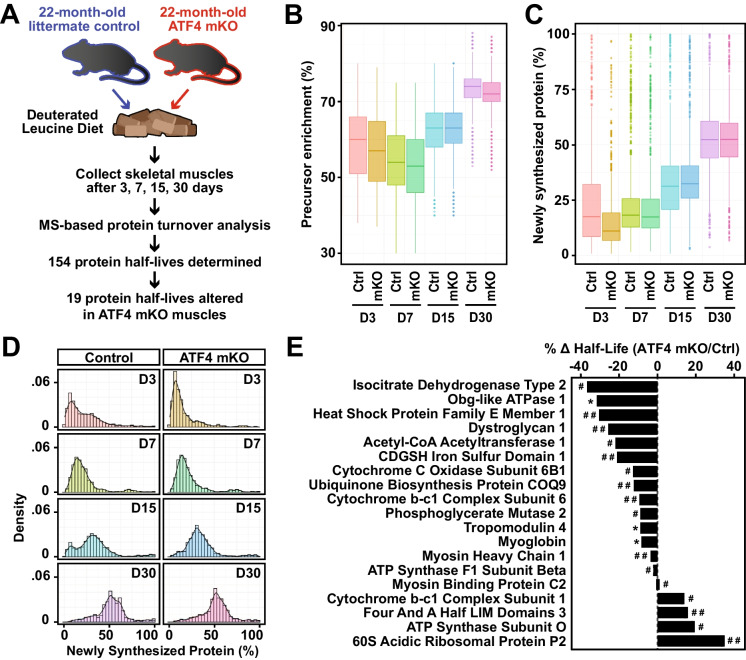
ATF4 influences turnover of specific proteins in skeletal muscle. **A** Cohorts of 22-month-old littermate control and ATF4 mKO mice were provided a standard chow diet containing deuterated leucine for 3, 7, 15, or 30 days before collection of skeletal muscle for MS-based protein turnover analysis. The half-lives of 154 skeletal muscle proteins were determined, and 19 of those proteins were found to have altered half-lives in ATF4 mKO muscles. Data are from 3 mice per timepoint and genotype. **B** Precursor pool enrichment of heavy leucine in control and ATF4 mKO muscles at each timepoint. **C** Percentage of newly synthesized proteins in control and ATF4 mKO muscles. **D** Distribution of percentage of newly synthesized protein in control and ATF4 mKO muscles. **E** Proteins whose turnover rates were significantly different in ATF4 mKO muscles (*P* ≤ 0.05), and the percent change in their half-lives; *q value < 0.05, ^#^*q* value < 0.1, and ^##^*q* value < 0.25

Through complex regulatory mechanisms that are not yet well understood, aging alters protein turnover in many tissues, including skeletal muscle [13, 29, 61–63]. Furthermore, age-related disruptions in protein turnover are thought to play an important role in age-related declines in cellular function [64–67]. Because loss of ATF4 expression reduced age-related muscle atrophy and weakness and altered the expression of many transcripts involved in translational control and protein synthesis, we hypothesized that loss of ATF4 expression might alter protein turnover in aged skeletal muscle. To test this hypothesis, we fed a deuterated leucine diet to 22-month-old control and ATF4 mKO mice, collected skeletal muscles after 3, 7, 15, and 30 days on the deuterated leucine diet, and then subjected the skeletal muscles to a recently developed mass spectrometry-based method that quantifies turnover of the most highly abundant skeletal muscle proteins (Fig. 6A [26]). Deuterated leucine was efficiently incorporated into skeletal muscle proteins, with ~ 75% of all leucine in the muscles isotopically labeled at the 30-day timepoint (Fig. 6B). Furthermore, more than half of all measured proteins were greater than 50% newly synthesized by the 30-day timepoint, and there was no significant difference between the two genotypes (Figs. 6C–D).

The time course data from this study allowed us to quantitate the half-lives of 154 highly abundant skeletal muscle proteins in 22-month-old control and ATF4 mKO muscle (Fig. 6A and Table S5). Of these 154 proteins, 19 proteins (~ 12%) had significantly altered turnover rates in ATF4 mKO muscles (Fig. 6E, Fig. S6 and Table S5). Interestingly, most of these 19 proteins are known to have important roles in skeletal muscle metabolism and structure [68–85]. Furthermore, most of the proteins with differential half-lives in ATF4 mKO muscles had decreased half-lives, indicating higher rates of protein turnover (Fig. 6E). In addition, at least one of the proteins with a higher rate of protein turnover in aged ATF4 mKO muscles (isocitrate dehydrogenase 2) is known to acquire a lower turnover rate during skeletal muscle aging [63]. These results indicate that ATF4 expression in aged skeletal muscle fibers alters turnover of specific proteins with important metabolic and structural functions, with most of the regulated proteins having a slower turnover rate in the presence of ATF4.

## Discussion

**Fig. 7:**
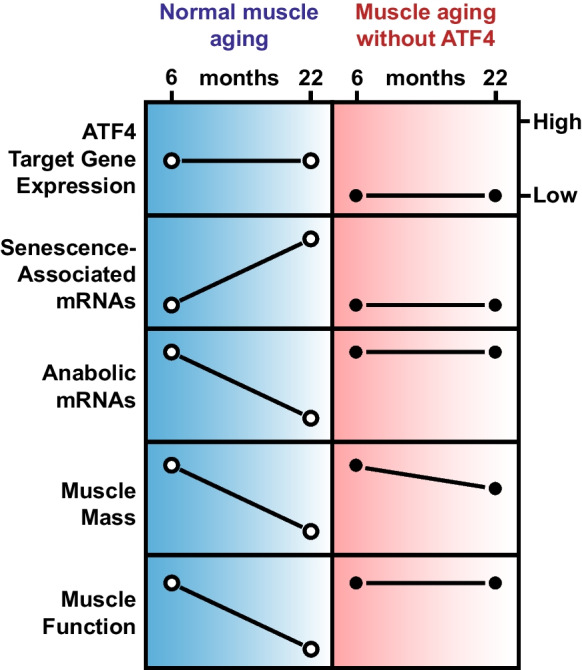
Summary of identified age-related changes in mouse skeletal muscle, in the presence and absence of ATF4 expression

In the current study, we aimed to better understand the role of ATF4 in skeletal muscle aging. Our data are summarized by the schematic in Fig. 7. During normal muscle aging, ATF4 mediates basal expression of approximately 30 direct target genes involved in stress signaling and translational control. Interestingly, basal expression of these 30 genes does not significantly change between 6 and 22 months of age. However, many other things do change between 6 and 22 months of age, including but not limited to induction of mRNAs involved in cellular senescence, repression of anabolic mRNAs involved in protein synthesis and mitochondrial function, and age-related declines in muscle mass, strength, muscle quality, and endurance exercise capacity. These are some of the characteristics of normal muscle aging.

When ATF4 is chronically removed from skeletal muscle fibers, as in ATF4 mKO mice, basal expression of the 30 ATF4 target genes is chronically reduced. This does not affect development of peak muscle mass or function, and thus, at 6 months of age, ATF4 mKO mice are phenotypically indistinguishable from littermate control mice. However, as ATF4 mKO mice become older, several differences emerge, including impaired induction of senescence-associated mRNAs, impaired repression of anabolic mRNAs, partial prevention of age-related skeletal muscle atrophy, and protection against age-related declines in strength, muscle quality, and endurance exercise capacity. Additionally, the phenotype of older ATF4 mKO muscles is accompanied by altered turnover of several proteins with crucial roles in skeletal muscle structure and metabolism. These data strongly suggest ATF4 as an important mediator of skeletal muscle aging.

One important unresolved question is whether ATF4 expression changes with aging. The current data indicate that *ATF4* mRNA levels do not increase and in fact, slightly decrease, in skeletal muscle between 6 and 22 months of age. However, ATF4 protein levels are primarily regulated by post-transcriptional mechanisms [6], so changes in the level of *ATF4* mRNA do not necessarily predict corresponding changes in the level of ATF4 protein. Unfortunately, we have been unable to quantify the level of ATF4 protein in skeletal muscle, either by immunoblot or mass spectrometry, presumably due to the low abundance and short half-life of ATF4, or a lack of highly sensitive and specific antibodies against ATF4. Thus, based on the available information, we cannot determine whether ATF4 protein levels change between 6 and 22 months of age, and due to this uncertainty, we did not include ATF4 levels in our model in Fig. 7. Additional studies and probably additional technological advances will be needed to determine whether ATF4 protein levels significantly change during skeletal muscle aging.

Skeletal muscle aging is a slowly progressive process that occurs over decades in humans and over many months in mice. In contrast to aging, acute stress conditions such as fasting and immobilization induce skeletal muscle atrophy quite rapidly, within days in humans and within hours in mice. Interestingly, ATF4 also mediates fasting-and immobilization-induced skeletal muscle atrophy [8, 9]. However, during those acute stress conditions, ATF4 target gene expression increases to levels that are substantially higher than the basal levels observed in this study, which utilized 6- and 22-month-old mice that were neither fasted nor immobilized. These considerations could suggest a potential model in which strong induction of ATF4 target genes during acute stress conditions leads to rapid loss of skeletal muscle mass and function, whereas chronic basal expression of ATF4 target genes during natural aging promotes a slow erosion of skeletal muscle mass and function. Another potential model, not mutually exclusive with the first, is that specific detrimental activities of ATF4 are preferentially activated with age, perhaps mediated by differential abundance of a specific binding partner in aged muscle. Indeed, ATF4 is not a standalone transcription factor capable of activating genes by itself, as its name implies, but rather, ATF4 is one half of many possible heterodimeric transcription factors, each with unique and highly context-dependent functions [6]. In mouse skeletal muscle fibers, ATF4 forms multiple heterodimers, but only one ATF4 heterodimer, composed of ATF4 and C/EBPβ, appears to mediate muscle atrophy [33]. From this perspective, it is interesting to note that aging significantly increases the level of *C/EBPβ* mRNA by 2.5-fold in both control skeletal muscle (FDR 2.0 × 10^–23^) and ATF4 mKO muscle (FDR 2.5 × 10^–9^) (Table S1). These data, coupled with the phenotype of the ATF4 mKO mice, suggest a potential model in which aging promotes formation of the ATF4-C/EBPβ heterodimer in skeletal muscle (at least partly through an ATF4-independent increase in C/EBPβ expression), and the ATF4-C/EBPβ heterodimer promotes a loss of muscle mass and function between 6 and 22 months of age. This will be an important area for future investigation.

Other important areas for future investigation include the downstream mechanisms (ATF4 target genes) that mediate age-related skeletal muscle atrophy and the upstream mechanisms that control ATF4 protein levels in skeletal muscle fibers. Two potentially important downstream mediators are the *p21/Cdkn1a* and *Grb10* genes, which encode negative regulators of skeletal muscle mass [10, 50–53] and exhibit chronically reduced expression in ATF4 mKO muscles at both 6 and 22 months of age. It also remains possible that additional ATF4 target genes may become active between 6 and 22 months and contribute to age-related changes. As an example, one ATF4 target gene that promotes muscle atrophy, *Gadd45a* [9, 33], had lower expression in ATF4 mKO muscles at 22 months, but not 6 months (Table S1). We suspect that multiple ATF4 target genes may be involved in age-related skeletal muscle atrophy, but further studies will be needed to test this hypothesis. There are also several potential upstream regulators of ATF4 protein expression during muscle aging. For example, in other cell types, ATF4 protein levels can be increased by mTORC1 signaling, the integrated stress response, and other signaling pathways that reduce eIF2B activity, all of which have been implicated in skeletal muscle aging [86–89]. Furthermore, pharmacologic inhibitors of the integrated stress response and mTORC1 have been proposed as potential approaches for the prevention and treatment of age-related skeletal muscle atrophy and weakness [90–92], and ursolic acid and tomatidine, two small molecules that reduce age-related skeletal muscle atrophy and weakness in mouse models, also reduce ATF4-mediated gene expression in aged skeletal muscle, consistent with the phenotype of ATF4 mKO mice [11, 14, 15]. A better understanding of these upstream and downstream mechanisms could inform therapeutic approaches.

Interestingly, ATF4 activity is positively associated with lifespan and healthspan in a number of preclinical models [93, 94], although much of this work has focused on liver. This raises the question of how ATF4 activity can be positively associated with geroprotective interventions in some contexts and also play a detrimental role in other contexts, such as skeletal muscle aging. We speculate that the complexity of ATF4-dependent phenotypes during aging is largely if not entirely due to the complexity of ATF4 itself. As discussed above, ATF4 is a rate-limiting component of many different heterodimeric bZIP transcription factors with unique and highly context-dependent functions [6]. Thus, ATF4 is a multifunctional protein that can participate in a wide range of biological processes, some of which may be geroprotective, and some of which may be degenerative.

Although we believe the results of this study are interesting and important, we also believe that the study has some limitations. First, mice have advantages but also inherent limitations as a model system for studying skeletal muscle aging, so it will be important to extend these studies to other species, particularly humans. Second, the transcriptomic and proteomic data presented here are correlative and do not establish causal mechanisms beyond implicating ATF4 in a variety of age-related processes. Furthermore, the slow nature of aging studies precludes us from quickly testing the suggested hypotheses. Thus, additional studies will be needed to determine which downstream effects of ATF4 are essential for age-related skeletal muscle atrophy and weakness. Third, the current study only investigated mice up to 22 months of age, so we do not yet know whether ATF4 plays an essential role in later stages of muscle aging, which are characterized by more profound muscle atrophy and functional deficits. Fourth, the current study examined the role of one transcription regulatory protein, ATF4, in one genetic background (C57BL/6), one gender (males), and a few muscle types. However, many other factors also influence age-related loss in muscle mass and function, including motoneuron loss, neuroendocrine factors, genetic background, gender, and inherent properties of specific muscle types and specific muscle fiber types [3, 95]. Thus, it will be important in future studies to investigate how ATF4 interacts with other pathogenic processes and mediators (such as motoneuron loss and neuroendocrine factors), whether ATF4 might also play a role in age-related loss in muscle mass and function in different contexts, (including other genetic backgrounds, female gender, and other muscle types) and whether ATF4 has fiber type-specific effects.

An additional potential limitation of the current study relates to the non-natural *ATF4-Cacna1i* fusion transcript and decreased levels of *Rps19bp1* mRNA in ATF4 mKO muscles. These may be off-target effects of Cre-mediated excision of *ATF4* exons 2 and 3, and similar changes (lower levels of *Rps19bp1* mRNA and an increased level of a *Cacna1i*-assigned transcript) have also been observed in the livers of liver-specific ATF4 knockout mice [54] and in global ATF4 knockout mice and cell lines with constitutive loss of *ATF4* exons 2 and 3 [96, 97]. Because *Rps19bp1* mRNA is poorly expressed in control skeletal muscle and only trace amounts of the *ATF4-Cacna1i* fusion transcript are observed in ATF4 mKO muscles (Fig. S3), it seems unlikely that changes in these transcripts could explain the ATF4 mKO phenotype. Furthermore, the phenotypes observed in ATF4 mKO muscles are consistent with previous observations that RNAi-mediated knockdown of ATF4 mRNA reduces muscle atrophy, and ATF4 overexpression induces muscle atrophy [8].

In summary, the current study identifies ATF4 as a likely mediator of several age-related changes in skeletal muscle, including repression of genes involved in mitochondrial function and protein synthesis, induction of genes involved in cellular senescence, and most importantly, age-related declines in muscle mass, strength, muscle quality, and endurance exercise capacity. These findings could contribute to a greater mechanistic understanding of skeletal muscle aging and inform development of new approaches to preserve muscle mass and function in older adults.

## Supplementary information

ESM 1. (PDF 118 kb)

Supplementary Fig 1. ATF4 contributes to age-related declines in skeletal muscle strength, muscle quality, and endurance exercise capacity. Weight-matched cohorts of 6- and 22-month-old littermate control and ATF4 mKO mice were subjected to assessments of total body weight (A), in vivo grip strength (B), ex vivo specific force (C) and in vivo treadmill running (D). Each data point represents one mouse, and horizontal bars denote means. The grip strength and specific force data from 22-month-old control and ATF4 knockout mice were shown previously [11]. P-values compare control and ATF4 mKO mice at each time point using two-way ANOVA with Šidák’s multiple comparisons test. (EPS 1794 kb)

Supplementary Fig 2. ATF4 is necessary for age-related skeletal muscle atrophy. Weight-matched cohorts of 6- and 22-month-old littermate control and ATF4 mKO mice were subjected to assessments of combined weight of bilateral gastrocnemius muscles (A), combined weight of bilateral quadriceps muscles (B) and average quadriceps muscle fiber diameter (C). Each data point represents one mouse, and horizontal bars denote means. P-values compare control and ATF4 mKO mice at each time point using two-way ANOVA with Šidák’s multiple comparisons test. (EPS 1689 kb)

Supplementary Fig 3. Effect of *ATF4* exon 2-3 excision on the *Rps19bp1* and *Cacna1i* genes in skeletal muscle. A, Schematic of the floxed *ATF4* allele in littermate control mice, which lack Cre recombinase. The *ATF4* gene has 3 exons, and the coding region is contained in exons 2 and 3. In the floxed *ATF4* allele, LoxP sites lie upstream of *ATF4* exon 2 and downstream of *ATF4* exon 3. The *Rps19bp1* gene contains 4 exons, lies approximately 3 kb from the 3’ end of *ATF4* exon 3, and is transcribed from the opposing strand of DNA. The *Cacna1i* gene contains 37 exons, lies approximately 30 kb from the 3’ end of *ATF4* exon 3, and is transcribed from the same strand of DNA as the *ATF4* gene. B, Schematic of the floxed *ATF4* allele in ATF4 mKO mice, which express Cre recombinase under control of the *MCK* promoter. In ATF4 mKO mice, Cre recombinase excises *ATF4* exons 2 and 3 in skeletal muscle fibers, leaving *ATF4* exon 1 in place. At a frequency that appears to be quantitatively low based on RNA-Seq read alignments (discussed below), some residual *ATF4* exon 1 is transcribed into the *Cacna1i* gene, ultimately generating a non-natural fusion transcript in which *ATF4* exon 1 (5’ untranslated region of the *ATF4* gene) is spliced to *Cacna1i* exon 2. C, RNA-seq read alignments in littermate control and ATF4 mKO skeletal muscle, with the Y-scale set at 0-1100 reads. In littermate control muscle, mRNA from the floxed *ATF4* allele is expressed at wild-type levels and easily detectable, but not highly abundant relative to major skeletal muscle transcripts. In ATF4 mKO muscle, *ATF4* mRNA (including sequence from *ATF4* exons 1-3) is dramatically reduced. At this scale, *Rps19bp1* mRNA and the *ATF4*-*Cacna1i* fusion transcript can be only faintly detected in littermate control muscle and ATF4 mKO skeletal muscle. D, RNA-seq read alignments in littermate control and ATF4 mKO skeletal muscle, with the Y-scale magnified to 0-80 reads. At this magnified scale, the level of *ATF4* mRNA greatly exceeds the upper limit in control muscle and is near the upper limit in ATF4 mKO muscle. In addition, at this magnified scale, a low level of *Rps19bp1* mRNA is apparent in control muscle and reduced in ATF4 mKO muscle, and a low level of the *ATF4*-*Cacna1i* fusion transcript is apparent in ATF4 mKO muscle. (EPS 4509 kb)

Supplementary Fig 4. ATF4 expression in skeletal muscle fibers contributes to age-related induction of transcripts involved in inflammation, cellular senescence, and Rho GTPase signaling. Heat map of Reactome gene sets that significantly increase in control muscles but not ATF4 mKO muscles between 6 and 22 months of age. (EPS 1704 kb)

Supplementary Fig 5. ATF4 expression in skeletal muscle fibers contributes to age-related repression of transcripts involved in mitochondrial function, metabolism of amino acids, polyamines, glutathione and nicotinamide, and protein synthesis. Heat map of Reactome gene sets that significantly decrease in control muscles but not ATF4 mKO muscles between 6 and 22 months of age. (EPS 1682 kb)

Supplementary Fig 6. ATF4 influences turnover of specific proteins in skeletal muscle. Raw regressions of the fraction of newly synthesized protein for proteins with significantly altered turnover rates in ATF4 mKO muscles relative to control littermates. Symbols represent individual peptide measurements for the specified protein at the indicated time-points. Circles represent control and triangles represent ATF4 mKO mice. Linear modeling statistics were applied to determine if the interaction between the log-transformed percent newly synthesized values and time are different between ATF4 mKO and control samples, and the unadjusted P-values of the difference in interaction are shown. (EPS 7526 kb)

Supplementary Table 1. RNAs from 6-month and 22-month-old ATF4 mKO and littermate control mouse tibialis anterior muscles as assessed by RNA-seq (20,425 unique transcripts). (XLSX 8132 kb)

Supplementary Table 2. Reactome gene set enrichment analysis (GSEA) of ATF4 mKO and littermate control muscles at 6 and 22 months of age. (XLSX 72 kb)

Supplementary Table 3. Transcripts induced by aging in control (FDR<0.1) but not ATF4 mKO muscles (711 unique transcripts). (XLSX 312 kb)

Supplementary Table 4. Transcripts repressed by aging in control (FDR<0.1) but not ATF4 mKO muscles (708 unique transcripts). (XLSX 327 kb)

Supplementary Table 5. Protein turnover rates in 22-month-old ATF4 mKO and littermate control muscles. (XLSX 34 kb)

## Declarations

SME, CMA, BBR, and ARJ are shareholders in Emmyon, Inc., which holds patent rights for uses of ursolic acid and tomatidine in skeletal muscle health. SME and CMA serve as officers at Emmyon, Inc., and BBR and ARJ serve as consultants.
