# Supplementary material for: The transcription regulator ATF4 is a mediator of skeletal muscle aging
Source: GeroScience. 2023 Apr 4;45(4):2525–43. doi: 10.1007/s11357-023-00772-y (PMC10071239; doi:10.1007/s11357-023-00772-y)
Supplement: Supplementary file 1 — (PDF 118 kb) [file 11357_2023_772_MOESM1_ESM.pdf]

SUPPLEMENTAL EXPERIMENTAL PROCEDURES

Protein turnover studies and mass spectrometric analysis

At 19.5 months of age, cohorts of control and ATF4 mKO mice were switched from standard chow (Harlan-Teklad formula 7913) to "Amino Acid Defined" chow (Envigo TD.99366), which contains 11.1 g/kg leucine. Ten weeks later, at 22 months of age, mice were switched to a modified Envigo TD.99366 diet that contained 11.1 g/kg deuterated leucine ([5,5,5-$^2H_3$]-L-leucine) in place of unlabeled leucine. Quadriceps muscles (3-5 per genotype and time point) were collected after 3, 7,15 or 30 days on the deuterated leucine diet, then stored in liquid nitrogen. Homogenization was performed by placing the frozen samples in 2mL safe lock bead-beating tubes (#022363352, Eppendorf, Hamburg, Germany) containing a lysis buffer composed of 8M Urea (#29700, Thermo Fisher Scientific, Waltham, MA) and 1X Protease/phosphatase inhibitor cocktail (#78446, Thermo Fisher Scientific, Waltham, MA) in 50 mM triethylammonium bicarbonate buffer (#T7408, Sigma Aldrich, St. Louis, MO) and a Qiagen stainless steel 5mm bead (#69989, Qiagen, Hilden, Germany). Samples were homogenized in a TissueLyzer II (Qiagen, Hilden, Germany) at 4°C three times for 3 minutes at 25Hz. Samples were subsequently sonicated 2 times for 15 seconds on a model CL-18 probe sonicator (Thermo Fisher Scientific, Waltham, MA) at 30% amplitude. Samples were spun at 15,000 x G for 15 minutes at 4°C to clear the lysate, and supernatants were transferred into new tubes. Protein quantitation was then performed using a BCA Protein Assay Kit (#23225, Pierce, Waltham, MA). Samples were processed and digested using a Suspension-traps (S-Traps) procedure (#C02-micro-80, Protifi, Huntington, NY) according to the manufacturer protocol. Aliquots of each sample containing 100 μg protein were brought to equal volumes with 50 mM triethylammonium bicarbonate buffer at pH 8 and SDS was added to a final concentration of 4%. The mixtures were reduced with 20 mM DTT (50°C for 10 minutes), then alkylated with 40 mM iodoacetamide (30 minutes at room temperature in the dark). Samples were acidified at with 12% phosphoric acid for final concentration of 1.2%. Samples were diluted with seven times the sample volume of S-Trap buffer, mixed, and transferred to S-Trap spin columns. Samples were spun through the column, washed, and bound peptides were digested on-column at 47°C for 1 hour, and at 37°C overnight with sequencing grade trypsin (Promega, San Luis Obispo, CA) at a 1:25 enzyme:substrate ratio (wt/wt). Peptides were then eluted into new tubes, dried in a SpeedVac, and reconstituted in 0.2% formic acid in water. Peptide supernatants were desalted with Oasis HLB 30-mg Sorbent Cartridges (Waters, Milford, MA; #186003908), concentrated, and resuspended in a solution containing mass spectrometric "Hyper Reaction Monitoring" retention time peptide standards (HRM, Biognosys, Schlieren, Switzerland; #Kit-3003) and 0.2% formic acid in water. Samples were analyzed by reverse-phase HPLC-ESI-MS/MS using the Eksigent Ultra Plus nano-LC 2D HPLC system (Dublin, CA) combined with a cHiPLC system directly connected to an orthogonal quadrupole time-of-flight SCIEX TripleTOF 6600 mass spectrometer (SCIEX, Redwood City, CA). Typically, mass resolution in precursor scans was approximately 45,000, while fragment ion resolution was approximately 15,000 in "high sensitivity" product ion scan mode. After injection, peptide mixtures were transferred onto a C18 pre-column chip (200 μm × 6 mm ChromXP C18-CL chip, 3 μm, 300 Å; SCIEX, Redwood City, CA) and washed at 2 μL/minute for 10 minutes with the loading solvent (H_2O/0.1% formic acid) for desalting. Peptides were transferred to the 75 μm × 15 cm ChromXP C18-CL chip, 3 μm, 300 Å (SCIEX, Redwood City, CA) and eluted at 300 nL/minute with a 3-hour gradient using aqueous and acetonitrile solvent buffers for both the data dependent acquisition (DDA) and the data independent acquisition (DIA). Solvents were prepared as follows: mobile phase A, 2% acetonitrile/98% of 0.1% formic acid (vol/vol) in water; mobile phase B, 98% acetonitrile/2% of 0.1% formic acid (vol/vol) in water. All samples were analyzed by DDA for downstream analysis of protein turnover. After acquisition of approximately five samples, TOF MS spectra and TOF MS/MS spectra were automatically calibrated during dynamic LC-MS and MS/MS autocalibration acquisitions injecting 25 fmol β-galactosidase. For collision-induced dissociation tandem MS (CID-MS/MS), the mass window for precursor ion selection of the quadrupole mass analyzer was set to ±1 m/z. The precursor ions were fragmented in a collision cell using nitrogen as the collision gas. Advanced information-dependent acquisition (IDA) was used for MS/MS collection on the TripleTOF

6600 to obtain MS/MS spectra for the 30 most abundant parent ions following each survey MS1 scan (allowing typically for 100 ms acquisition time per each MS/MS). Dynamic exclusion features were based on value M not m/z and were set to an exclusion mass width of 50 mDa and an exclusion duration of 30 seconds. Mass spectrometry DDA raw files were database searched using Protein Pilot Software 5.0 (revision 4769, Paragon Algorithm 5.0.0.0.4767, SCIEX). All data files were searched using the UniProt Mus Musculus Proteome (downloaded December 2016). The following sample parameters were used in Protein Pilot: sample type set to SILAC (Leu +3), trypsin digestion, cysteine alkylation set to iodoacetamide, urea denaturation, and acetylation emphasis. Processing parameters were set to "Biological modification," and a thorough ID search effort was used. A global FDR of 1% was chosen using the Protein Pilot FDR analysis tool. All samples were also analyzed by DIA analysis for downstream analysis of protein abundance changes. The DIA acquisition method utilized variable precursor isolation window width acquisitions [1]. In these DIA acquisitions, windows of variable width (5 to 90 m/z) were passed in incremental steps over the full mass range (m/z 400–1,250). The cycle time of 3.2 seconds includes a 250-ms precursor ion scan followed by a 45-ms accumulation time for each of the 64 DIA segments. The variable windows were determined according to the complexity of the typical MS1 ion current observed within a certain m/z range using a SCIEX "variable window calculator" algorithm (more narrow windows were chosen in "busy" m/z ranges, wide windows in m/z ranges with few eluting precursor ions) [2]. DIA tandem mass spectra produce complex MS/MS spectra, which are a composite of all the analytes within each selected Q1 m/z window. All collected data were processed in Spectronaut using a sample-specific library generated by DDA analysis of the same samples using the settings described above.

Quantitative analysis of abundance and turnover

For calculation of protein abundance changes, DIA acquisitions from six samples (three ATF4 mKO and three littermate control samples) were quantitatively processed using Spectronaut v14 (14.7.201007) software from Biognosys (Schlieren, Switzerland). A sample-specific spectral library was used for Spectronaut processing of the DIA data. Spectronaut Identification-Decoy settings were set to scrambled. Quantification of peptide level results was based on the sum of the top 1-10 precursors, and protein level quantification was based on the sum of the top 1-7 peptides. Data filtering was set to q-value sparse with no data imputation. Cross run normalization was turned on, with q-value sparse row selection and a local normalization strategy. In the workflow settings, profiling strategy was set to iRT profiling with row selection based on a q-value < 0.01. All other Spectronaut settings were set to the BGS Factory Settings. Relative quantification was performed comparing different conditions (ATF4 mKO vs control) to assess fold changes. Changes in protein abundance with q-values (FDR-controlled) less than 0.05 were determined to be significant. Annotations and full quantitative changes in protein abundance are provided in Table S5. Quantitative MS1 peak areas were extracted from DDA raw files in the Skyline-Daily software platform. ProteinPilot search results were imported into the Skyline software to build a spectral library, followed by import of raw MS files for extraction of chromatographic peak areas. Files were processed retention time alignment calculated based on iRT peptides (Biognosys, Switzerland) and peak scoring using the mProphet model. The 'permute isotope modification' function was used to populate Skyline with isotopologues of peptides containing leucine, which are required because isotopologue peak area ratios are required to calculate precursor enrichment and protein turnover rates in downstream analysis. A custom skyline report containing all peptide and protein characteristics, annotations, and quantitative information including isotopologue peak areas was exported and used for downstream analysis and calculation of protein turnover rates in R using in-house R scripts available on GitHub (https://github.com/CameronWehrfritz/Adams-Protein-Turnover-Paper.git). Precursor-pool corrected protein turnover rates were calculated in R using the same approach employed in previous studies using the Topograph software platform [3-5]. Prior to calculating the relative isotope enrichment (RIA) of the amino acid precursor pool, peptide isotope distribution deconvolution was performed to normalize for signal contributed by naturally occurring heavy isotopes. For every isotopologue of a leucine containing

peptide, a theoretical isotope distribution was generated by calculating list of m/z bin intensities for all possible isotope peaks expected to contribute at least 1% of the total isotope distribution signal. The observed isotopologue envelopes for each leucine containing peptide were determined based on the relative peak areas of each isotope peak integrated by Skyline. The amount of each isotopologue is the observed distribution of isotope peaks was determined by calculating the fractional amount of each theoretical isotopologue distribution that best fit the observed signal using a skewed least squared analysis previously described [6,7]. The calculated relative abundances of peptide isotopologues from peptides containing two or more leucines was then used to calculate the RIA of the amino acid precursor pool using methods previously described [8,9]. For peptides containing more than one leucine, the theoretical isotopologue distribution generated by any RIA is unique. Therefore, the isotopologue patterns from these peptides were used to back-calculate the enrichment of heavy leucine in the amino acid precursor pools. We used a similar strategy described by Hsieh et al [3] to solve the precursor enrichment for all peptides containing 2-5 leucines and used the median precursor enrichment calculated across all peptides as the precursor enrichment for downstream estimation of newly synthesized protein. Precursor RIA was calculated separately for each genotype and timepoint. The precursor pools did not significantly change between treatment groups but increased over timepoints with the incorporation of heavy leucine from the diet (Fig. 6B). Median precursor pool enrichments were then applied to re-calculate the fractional abundances of newly synthesized peptides vs pre-existing peptides and calculating a percentage of newly synthesized peptide. For each protein, turnover rate was calculated by fitting the percentage of newly synthesized peptide of all its unique peptides to a first-order exponential decay and solving for the rate constant. The distribution of newly synthesized proteins did not significantly differ between ATF4 mKO and littermate control muscles but increased over time as expected (Fig. 6C-D). This yielded the protein turnover rates that were transformed into half-lives using a simple conversion [half-life = -ln(2)/turnover]. For statistical comparison of turnover rates between ATF4 mKO and control samples, first order equations were natural log transformed, making a linear relationship between the log of percent newly synthesized proteins and time. Then linear modeling statistics were applied to determine if the interaction between the log-transformed percent newly synthesized values and time are different between ATF4 mKO and control samples, and the p-value of the difference in interaction was used to determine whether protein turnover rates were significantly different by genotype. A full report of protein turnover rates, annotations, variance, statistical analysis, and other quantitative information is provided in Table S5.

REFERENCES

1. Collins, B. C., Hunter, C. L., Liu, Y., Schilling, B., Rosenberger, G., Bader, S. L., Chan, D. W., Gibson, B. W., Gingras, A. C., Held, J. M., Hirayama-Kurogi, M., Hou, G., Krisp, C., Larsen, B., Lin, L., Liu, S., Molloy, M. P., Moritz, R. L., Ohtsuki, S., Schlapbach, R., Selevsek, N., Thomas, S. N., Tzeng, S. C., Zhang, H., and Aebersold, R. (2017) Multi-laboratory assessment of reproducibility, qualitative and quantitative performance of SWATH-mass spectrometry. *Nat Commun* **8**, 291. https://doi.org/10.1038/s41467-017-00249-5
2. Schilling, B., Gibson, B. W., and Hunter, C. L. (2017) Generation of High-Quality SWATH(®) Acquisition Data for Label-free Quantitative Proteomics Studies Using TripleTOF(®) Mass Spectrometers. *Methods Mol Biol* **1550**, 223-233. https://doi.org/10.1007/978-1-4939-6747-6_16
3. Hsieh, E. J., Shulman, N. J., Dai, D. F., Vincow, E. S., Karunadharma, P. P., Pallanck, L., Rabinovitch, P. S., and MacCoss, M. J. (2012) Topograph, a software platform for precursor enrichment corrected global protein turnover measurements. *Mol Cell Proteomics* **11**, 1468-1474. https://doi.org/10.1074/mcp.O112.017699
4. Basisty, N., Dai, D. F., Gagnidze, A., Gitari, L., Fredrickson, J., Maina, Y., Beyer, R. P., Emond, M. J., Hsieh, E. J., MacCoss, M. J., Martin, G. M., and Rabinovitch, P. S. (2016) Mitochondrial-targeted catalase is good for the old mouse proteome, but not for the young: 'reverse' antagonistic pleiotropy? *Aging Cell* **15**, 634-645. https://doi.org/10.1111/acel.12472

5. Basisty, N., Meyer, J. G., and Schilling, B. (2018) Protein Turnover in Aging and Longevity. *Proteomics* **18**, e1700108. https://doi.org/10.1002/pmic.201700108
6. Jennings, M. E., 2nd, and Matthews, D. E. (2005) Determination of complex isotopomer patterns in isotopically labeled compounds by mass spectrometry. *Anal Chem* **77**, 6435-6444. https://doi.org/10.1021/ac0509354
7. Brauman, J. I. (1966) Least Squares Analysis and Simplification of Multi-Isotope Mass Spectra. *Analytical Chemistry* **38**, 607-610. https://doi.org/10.1021/ac60236a020
8. Doherty, M. K., Whitehead, C., McCormack, H., Gaskell, S. J., and Beynon, R. J. (2005) Proteome dynamics in complex organisms: using stable isotopes to monitor individual protein turnover rates. *Proteomics* **5**, 522-533. https://doi.org/10.1002/pmic.200400959
9. Hellerstein, M. K., and Neese, R. A. (1992) Mass isotopomer distribution analysis: a technique for measuring biosynthesis and turnover of polymers. *Am J Physiol* **263**, E988-1001. https://doi.org/10.1152/ajpendo.1992.263.5.E988
